# Markov Blankets and Mirror Symmetries—Free Energy Minimization and Mesocortical Anatomy

**DOI:** 10.3390/e26040287

**Published:** 2024-03-27

**Authors:** James Wright, Paul Bourke

**Affiliations:** 1 Centre for Brain Research, and Department of Psychological Medicine, School of Medicine, University of Auckland, Auckland 1010, New Zealand; 2 School of Social Sciences, Faculty of Arts, Business, Law and Education, University of Western Australia, Perth, WA 6009, Australia

**Keywords:** free energy principle, active inference, predictive coding, Markov blankets, cortical development, cortical mesoanatomy, cortical self-organization

## Abstract

A theoretical account of development in mesocortical anatomy is derived from the free energy principle, operating in a neural field with both Hebbian and anti-Hebbian neural plasticity. An elementary structural unit is proposed, in which synaptic connections at mesoscale are arranged in paired patterns with mirror symmetry. Exchanges of synaptic flux in each pattern form coupled spatial eigenmodes, and the line of mirror reflection between the paired patterns operates as a Markov blanket, so that prediction errors in exchanges between the pairs are minimized. The theoretical analysis is then compared to the outcomes from a biological model of neocortical development, in which neuron precursors are selected by apoptosis for cell body and synaptic connections maximizing synchrony and also minimizing axonal length. It is shown that this model results in patterns of connection with the anticipated mirror symmetries, at micro-, meso- and inter-arial scales, among lateral connections, and in cortical depth. This explains the spatial organization and functional significance of neuron response preferences, and is compatible with the structural form of both columnar and noncolumnar cortex. Multi-way interactions of mirrored representations can provide a preliminary anatomically realistic model of cortical information processing.

## 1. Introduction

This paper argues that the free energy principle can be used to derive a model of neocortical self-organization accounting for anatomical structure and function at millimetric (mesoanatomical) scale. 

Embryologically the neocortex develops in accord with the “structural model” [1,2,3,4]. Neuron precursors migrate and differentiate under genetic control along radial pathways from archi- and paleocortical precursors, the pathways of migration partly determining later functional connections between neocortex and subcortical systems [5,6]. As cellular differentiations proceed, cell connectivity also undergoes modification under the distance rule [7,8] which describes inter-areal connectivity as an approximation to shortest and locally dense pathways–an arrangement facilitating metabolic efficiency and rapid interactions in a “small world”. Actual anatomy is a compromise between the radial versus small world arrangements [9]. Ultimately the neocortex becomes a closed extended sheet, embracing the subcortical systems from which it has arisen, and organized in depth as a six-layered structure of mixed excitatory and inhibitory cells [10]; essentially two layers of cell bodies with other layers in which inputs are received and lateral axons–largely excitatory-spread over greater distances. Among these laterally spreading axonal connections the superficial patch system is prominent [11,12,13]. This is made up of patches of cells that make connections skipping from patch to neighbouring patches in several steps, and thus forming gridworks apparently organized to distribute information between cortical locales. 

Lateral organization is highly variable at mesoanatomical scale, although much effort has been made to systematize the appearances [14]. Some parts of the cortex-notably the primary visual cortex of large animals–are organized in a columnar fashion, in which zones of short axon neurons are surrounded by groups of superficial patch cells, creating macrocolumns, but elsewhere this organization is minimal to apparently absent. Yet the superficial patch system is ubiquitous, and is overlapping where columnar organization is absent [12]. Within macrocolumns individual cells exhibit organization according to the stimulus preferences of cells. Neurons that respond to straight line visual stimuli are organized about the center of macrocolumns, with orientation preference from 0–180 degrees circling the center from 0–360 degrees, creating a singularity [15]. Superficial patch cells have a tendency to link cells with common orientation preference in adjacent columns–“like to like” [16]. Cells also show orderly, but more complicated structured preferences for stimulus spatial and temporal frequencies [17]. In noncolumnar cortex neurons also show the same types of preference, but with minimal or no apparent order [18]. 

Puzzles surround the significance of columnar structure versus its absence, as well as the functional significance of the neuron preferences. In animals with columnar visual areas, organized orientation preferences are present at birth [19] without having required exposure to visual stimuli, yet structure is not sustained in later development if the animal is deprived of stimuli of any given orientation–cells with that preference being lost [20]. Particular difficulties surround the nature of ocular dominance columns. In some animals with binocular vision these are organized in stripes in parts of the visual cortex that receive inputs from both eyes. Orientation preferences surrounding their singularities form mirror reflections between side-by-side columns, and between singularities above and below in a single column [21]—exaggerating a tendency toward mirroring between adjacent singularities much less apparent in the monocular cortex. Inputs from both eyes are organized topographically, as a map of each eye’s visual field, with input from each eye alternating, column by column, with that of the other. This hints strongly of a locus in which comparisons can be made between each eye’s input, as required for binocular vision. However, not all animals with binocular vision have ocular dominance columns. New world owl monkeys provide a particularly difficult instance [22]. They have either very poorly ordered, or absent, ocular dominance columns–yet it can be shown that they too receive alternating inputs from their eyes as if dominance columns were present. These and other findings have led to suggestions that columns are “spandrels”-viz: geometric forms without necessary function [23]. The view taken in this paper is that the columns represent one end of a spectrum of orderliness, but that a single organizational order underlies all.

Many theoretical formulations have been devised to account for limited aspects of mesocortical organization, without any wide-ranging explanation having achieved universal acceptance. In contrast, the free energy principle and its relationship to the concept of prediction error minimization, as advanced by Friston and colleagues [24,25,26,27,28,29,30,31,32,33,34] proposes an overarching explanation for self-organizing systems, including brain function as a specific instance. It draws parallels between laws of nature from the principle of least action to the organization of artificial and real intelligence. A central concept is provided by Jaynes’ linking of the maximum entropy principle of optimum statistical information to the laws of thermodynamics, and, in a further step of unification, to Bayesian inference [26,27,35], so that these concepts are duals, each to the others. As an example of such a duality, the interactions of coupled modes in a dynamical system are equivalent to iterative models of grammar [36]. This means that a correct explanation of neuronal growth and dynamics is *de facto* an explanation of information processing–a relationship Friston terms “self-evidencing”; that neuronal and synaptic growth must tend inevitably toward a stable state in which perturbations created by inputs are predicted from earlier learning, and signals generated internally minimize the impact of uninformative current inputs on ongoing activity. This balancing control of information exchanges is supposed to take place both within the organism, and in its interactions with the environment. At an asymptotic limit (although never reached in life) a state of perfect adaptation to the environment is hypothetically attained. 

Another way to express this process is that within any system with a boundary via which it must interact with a surrounding environment, an open steady state must be reached in which equal and opposite signals are continuously exchanged via the boundary, so as to cancel each other. This boundary is termed by Friston a Markov blanket [37]. In neurophysiological terms this means at the asymptotic limit information exchanges between brain and environment would correspond exactly to their mutual information, and the variational free energy–effectively the uncertainty of the system–would be zero. A mapping of all sensory and motor interactions with the environment onto the structure of developed synaptic connectivity would have emerged, as pathways for neural signals replicating all the ways the organism has learned to interact with the world.

Prediction error minimization and free energy minimization within a canonical model of the cortical macrocolumn have been introduced within the structural model of the cortex [38,39]. Here we provide a broader account, showing how growth processes and the maintenance of excitatory/inhibitory balance result in a structure conforming to minimum free energy and prediction error minimization, and suggesting a functional unity underlies the paradoxical appearances in the anatomical findings outlined above.

This account is first formulated theoretically by considering the minimal properties that must emerge as the neural field self-organizes in accord with the free energy principle. We show that a particular anatomical order–one involving meso-scale mirror-symmetric systems of synaptic connection, and Markov blankets–ought to emerge during early development. Comparison is then made to outcomes of a biological growth model, itself matched to experimental data. There is agreement between the two models, and in combination they provide a provisional account of development and information processing in the neocortex.

## 2. Basic Considerations

### 2.1. Cells

Consistent with the structural model we assume that genetic determinants govern the pathways of cell migration in embryogenesis, ultimately leading to the characteristic six layered neocortex structure of excitatory and inhibitory neurons. Developing neurons operate close to metabolic limits imposed by their large surface area and high demands for ion pumping [40]. Synapses are few compared to the number of contacts made between axons and dendrites, so neurons form a sparse one-to-many network, with weak connectivity per synapse [41,42]. Neurons make synaptic contacts preferentially with neighbours, since dendritic and axonal trees are denser near their somas, but synaptic sparsity forces connection to jump intervening cells, so that closely placed neurons form densely interwoven and interpenetrating networks. 

### 2.2. Presynaptic Flux and Learning Rules

All neurons in the neural field exchange synaptic flux via all polysynaptic and monosynaptic routes to all other neurons. Peak synaptic flux delivered along all pathways of flow is given by

(1) $$\varphi_{ij}\left(t+\frac{\left|i-j\right|}{\nu }\right)=\varepsilon_{ij}g_{ij}\rho_{ij}Q_{j}\left(t\right)$$

where $\;\varphi_{ij}$ is the presynaptic flux received at the i−th neuron from the j−th neuron, $Q_{j}$ is the pulse rate of the j−th neuron, υ is the speed of signal spread, and $\frac{\left|i-j\right|}{\nu }$ is the delay from pulse generation to arrival of peak pulse density at presynapses, summed over all routes. Synaptic gains are separated into three time-scales, so that $\varepsilon_{ij},g_{ij},\rho_{ij}$ are the transient synaptic efficacy, the slow dynamic synaptic gain, and the structural synapse gains along the polysynaptic pathways, respectively. Synapses compete for resources on all three time-scales. The Hebbian gain terms in Equation (1) follow the unification of fast and slow synaptic learning rules proposed by Izhikevich and Desai [43] combining rapid modification of synapses by short-term plasticity (STP) and short-term depression (STD), with slower synaptic consolidation under the Bienenstock-Cooper-Monro (BCM) rule, including the slow “floating hook” limitation of consolidation by negative feedback. Following dendritic summation of presynaptic pulses, pulse generation follows a suitable sigmoid function—the details of which are inessential to the following arguments.

### 2.3. Excitatory/Inhibitory Balance

Homeostatic mechanisms keep the firing rates of cortical neurons and the balance of excitatory and inhibitory synaptic impulses within stable limits. Anti-Hebbian synaptic plasticity [44], the mechanisms of which are not yet fully understood, acts to normalize excitatory and inhibitory synaptic gains, while leaving the relative strengths of Hebbian influences unchanged. Competitive metabolic processes may mediate the anti-Hebbian effect, and, as observed experimentally, the time-course of anti-Hebbian plasticity is between hours and days. Yet rapid excitatory/inhibitory stabilization is essential, as attested by the ease with which epilepsy can be triggered by local cortical irritation or photic stimulation. The means by which ongoing rapid stabilization is maintained is key to our following arguments.

## 3. A minimum Free Energy Organizational Unit

### 3.1. Rationale

Equation (1) can be re-written as a state equation for the neural field, in matrix/vector form

(2) $$\Phi \left(t\right)=GDQ\left(t\right)$$

Φ(t) is a matrix of unidirectional pre-synaptic flows, G(t) is a matrix of aggregate presynaptic gains associated with each flow, D(t) is a delay matrix of axo-dendritic conduction times, and Q(t) is a vector of action potential pulse rates. 

$\Phi \left(t\right)$ can be decomposed into spatial eigenmodes (patterns of synchronous and bidirectionally symmetric flow), and asymmetric and variable fluxes coupling the spatial eigenmodes into time-varying patterns. $G\left(t\right)$ and D(t) are a description of synaptic strengths and cell positions—with Φ(t) leading the development of the growth of synapses and cells. By tracking development of Φ(t) we should arrive at descriptions of both neural system dynamics, and the associated mesoanatomical order created by the consolidation of synaptic connections. This is first formulated theoretically by considering the minimal properties that must emerge as the neural field self-organizes in accord with the free energy principle.

### 3.2. Constraints

Development is subject to three constraints:

Firstly, minimization of free energy:

(3) F=A−C →0

where F is variational free energy, A is total presynaptic flux autocorrelation, and C is total presynaptic flux cross-correlation. These terms might also be read as *Accuracy* minus *Complexity*—so that when variational free energy is minimized the activities of all members of $\left\{\varphi_{ij}(t)\right\}$ can be reduced to probability densities among them. This in turn is equivalent to optimization of Bayesian model evidence, and this is what is meant by the term *self-evidencing* [26,27,35].

Secondly, minimization of perturbation from steady state, equivalent to prediction error minimization:

(4) $$\Delta \Phi^{+}\left(t\right)-\Delta \Phi^{-}\left(t\right)\to 0$$

where $\Delta \Phi^{+}\left(t\right)$ and $\Delta \Phi^{-}\left(t\right)$ are each other’s negative vector sums. At asymptote, each acts to predict and minimize errors in the other. This constraint is here applied not only for sensory and motor exchanges, but to exchanges in the neural field at all levels. 

Thirdly, maintenance of excitatory/inhibitory balance:

(5) $$\sum \varphi_{e}\to \sum \varphi_{i}\to constancy$$

where $\sum \varphi_{e}$ is the total excitatory presynaptic flux, and $\sum \varphi_{i}$ is the total inhibitory presynaptic flux. This supplies a steady-state constraint that cell pulse rates remain stationary in the main, as development proceeds. 

### 3.3. Minimization of Free Energy, F→0

At each stage of growth, although there are an increasing number, n, of unidirectional flows of presynaptic flux as synaptic and cell numbers are increasing, the total autocorrelation, A, during a relatively short epoch, T, at all lags, τ, is

(6) $$A=\frac{1}{2T}\sum_{ij}^{n}\int_{0}^{T}\int_{-\infty }^{+\infty }\varphi_{ij}\left(t\right)\varphi_{ij}\left(t-\tau \right)d\tau dt$$

and for the $\frac{n}{2}$ pairs of bidirectional flows, total cross-correlation, C, is

(7) $$C=\frac{1}{T}\sum_{ij,ji}^{\frac{n}{2}}\int_{0}^{T}\int_{-\infty }^{+\infty }\varphi_{ij}\left(t\right)\varphi_{ji}\left(t-\tau \right)d\tau dt$$

Therefore free energy (flux autocorrelation and crosscorrelation have units of power, not energy, but in reference to open exchanges across a Markov blanket, are referred to as energies.) is zero when for all i,j,t,τ,

(8) $$\varphi_{ij}\left(t\right)\varphi_{ij}\left(t-\tau \right)+\varphi_{ji}\left(t\right)\varphi_{ji}\left(t-\tau \right)=\varphi_{ij}\left(t\right)\varphi_{ji}\left(t-\tau \right)+\varphi_{ji}\left(t\right)\varphi_{ij}\left(t-\tau \right)$$

Equation (8) describes ongoing variations of synaptic flux as the neural field interacts with imposed signals via a Markov blanket. On arithmetical grounds these variations require that at least one term $\varphi_{ij}$ is equal to a term $\varphi_{ji}$ at each time-step, creating trajectories about an absolute equilibrium. At this equilibrium all four terms are equal, energy is equipartitioned, excitatory or inhibitory fluxes between all pairs of cells are bidirectionally symmetrical, and the system is time-stationary and may be periodic. This equilibrium condition, when applied to all combinations of interactions between excitatory and inhibitory cells, corresponds to zero-lag synchronous oscillation [45]. For exchanges between pairs of excitatory cells, or between pairs of inhibitory cells, with τ=0, excitatory or inhibitory populations of neurons fire synchronously and exchange bidirectionally symmetrical flux. In exchanges between excitatory and inhibitory cells the inhibitory flux can be regarded as the negation of excitatory flux. Therefore, for $\tau =\left|i-j\right|/\nu$ (half the period of oscillation) excitatory and inhibitory cells fire in anti-phase with effectively the same symmetrical flux exchange. The collective effect of the exchanges is zero-lag synchronous oscillation, and under small perturbations the oscillating equilibrium is stable. Fields of synchrony are spatial eigenmodes of Φ(t). 

Conversely asymmetric exchanges of flux can mediate time-varying eigenmode couplings—that is, the control of perturbations about synchronous oscillation. The perturbations associated with eigenmode coupling necessarily become both minimal and efficient, analogous to the minimal pdV work in thermodynamic systems as pressure and temperature differentials settle to stability. Leading to:

### 3.4. Minimization of Prediction Error, $\Delta \Phi^{+}\left(t\right)-\Delta \Phi^{-}\left(t\right)\to 0$

Symmetric exchanges of flux at equilibrium meet the condition by definition. Asymmetric exchanges must evolve to become paired so that each one of a pair generates a flux oppositely directed to the other as closely as possible—yet these cannot be between the same cells. This requires dual systems of connection, one system the mirror image of the other, and since these connections must mediate coupling between spatial eigenmodes rather than simply blocking eigenmode interactions, the eigenmodes themselves must occur as duplicates with mirror-symmetry.

### 3.5. Excitatory/Inhibitory Balance, $\sum \varphi_{e}\to \sum \varphi_{i}\to Constancy$

Synchronous equilibrium itself requires equal exchange between excitatory and inhibitory neurons. If the entire field is to remain in balance, parts of the field in excess excitation must interact with parts of the field in excess inhibition, and vice-versa. Therefore each of the systems of coupled eigenmodes in a mirrored pair must interact with its partner to establish joint excitatory/inhibitory balance—necessarily requiring collision of travelling waves in the neural field—as follows. 

### 3.6. Interactions within and between Spatial Eigenmodes

**Figure 1:**
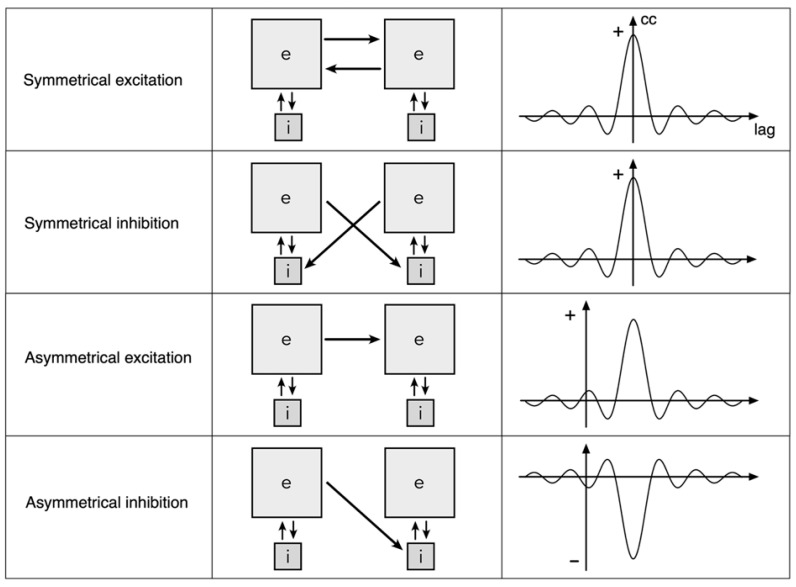
Exchanges between spatial eigenmodes. The grey squares marked e and i represent clusters of excitatory and inhibitory neurons whose interaction generates a field of synchronous oscillation (a spatial eigenmode). Bridging between the synchronous systems, excitatory presynapses link to either the excitatory cells or the inhibitory cells in the neighbouring assembly, and do so either symmetrically or asymmetrically. Approximate aggregate pulse cross-correlations between assemblies of excitatory cells in each of the paired eigenmodes are shown on the right.

Figure 1 shows the four possible ways that adjacent areas in a neural field, each area part of a spatial eigenmode organization, can interact. In the middle column blocks of interacting excitatory and inhibitory cells constituting spatial eigenmodes are shown cross connected by excitatory links. The cross-connections shown are those of medium or long-range connections, and are excitatory only. Short-range inhibitory cross-links would also be capable of mediating analogous effects to those next described, but are here ignored for simplicity. 

In the top row, interaction is symmetrical and excitatory, maximizing co-synchrony while increasing total excitation. In the second row interaction is symmetrical and inhibitory, in which case equal and opposite components in the colliding waves are cancelled, permitting co-synchrony with reduction of total excitation. Asymmetric interactions shown in the remaining two rows mediate eigenmode cross-coupling, with an increase or reduction in total excitation respectively.

### 3.7. Redundancy and Information Storage

The hypothetical mirror-symmetric connection systems require a 2:1 redundancy of the information storage in their synapses. Using the Nyquist and Shannon-Hartley theorems, and considering n directed synaptic couplings as unit-valued and composed of S synapses that have been shaped by learning, with the remainder considered random *ab initio*. Thus

(9) $$\frac{S}{n}=\frac{C}{A}\leq 1$$

is a synaptic signal/noise ratio, and

(10) $$D=n{log}_{2}\left(1+\frac{S}{n}\right)$$

is the number of bits needed to specify the information stored in the synaptic couplings. 

If the information input to the system is smaller by a factor M than the synaptic storage capacity, then any one of $2^{D}/M$ distinct inputs can be stored redundantly, as S/n→1, learning approaches asymptote, and free energy zero. This provides a further condition, M≥2, for the emergence of paired mirror symmetric systems.

### 3.8. Mirror Symmetric Fields and Markov Blankets

**Figure 2:**
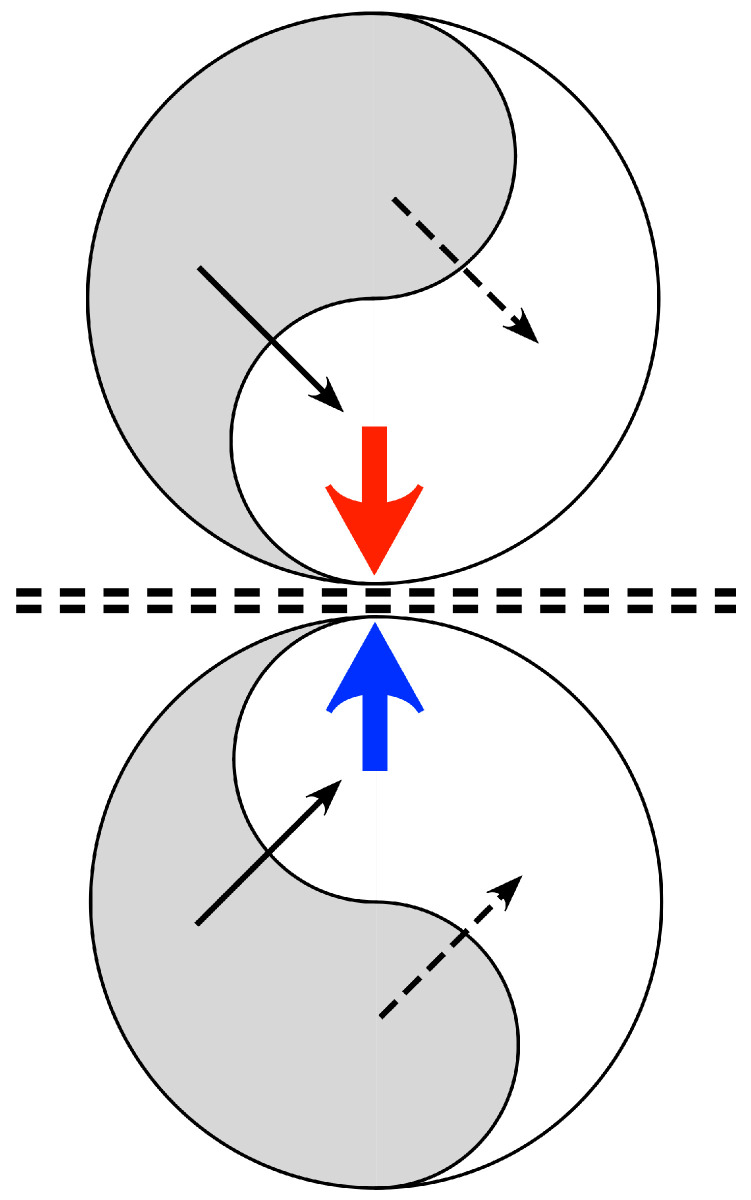
The topology of neural field interactions meeting requirements for minimization of free energy, minimization of prediction errors, and maintenance of excitatory/inhibitory balance. Paired mirror-symmetric systems of coupled spatial eigenmodes (arbitrarily represented as yin-yang figures) each interact internally via excitatory and inhibitory cross-couplings (solid and dashed black lines) generating oppositely directed travelling waves (colored arrows), that collide at the double dashed line.

Applying the mechanisms in Figure 1, Figure 2 illustrates a system composed of a pair of mirror-symmetric coupled spatial eigenmodes, each of the pair generating oppositely directed, colliding, travelling waves. The diagram shows the topology of the connections and flux exchanges—not a specific topography in the form shown. It is the synaptic connectivity that is essential, so the twin eigenmode systems might be separated by some distance, or their cell soma positions might be interdigitated. 

Excitatory/inhibitory stabilization can take place at the line of wave collision, since excess of excitation or of inhibition in waves from either side can be compensated at a fast time scale by shift between the symmetrical excitation and symmetrical inhibition modes of coupling-shifts modulated by the negative feedback “floating hook” property of the BCM rule, which diminishes synaptic gain in the more driven synapses. Adaptation may then be mediated more slowly by other cellular mechanisms of anti-Hebbian plasticity. As junctional exchange manages excitatory/inhibitory balance, prediction error minimization proceeds within each of the mirror duals, and free energy approaches zero. The signals arriving at the junction progressively maximize their mutual information. The mirror-like junction is therefore a Markov blanket, in Friston’s sense.

Interaction of dual systems can be generalized to multi-way interactions throughout the cortex, as continuously changing synaptic efficacies (Equation (1)) modulate and segregate the patterns of pulses and synaptic flux present at any one instant, achieving minimization of prediction errors in all exchanges. At a whole-brain scale, such a system is also suited to minimize prediction error in interaction with subcortical systems and the external milieu—thus forming a large-scale Markov blanket between cortex and subcortex. 

## 4. Emergence of Mirrored Synaptic Maps in Actual Anatomy

It must now be shown whether dual mirror-and-blanket systems can be identified in cortical mesoanatomy, and explained how their development takes place.

The preceding argument showed that as free energy is minimized, maximization of synchrony is a consequence. In biological terms the converse argument is more easily made with regard to the neocortex. Synchronous firing appears early in neuronal development along with the development of small world connectivity [46]. A substantial fraction of developing neurons succumb to apoptosis [47], and those neurons prevented from entering into synchrony succumb to apoptosis [48,49]. The surviving cells thus form a matrix maximizing synchronous oscillation. A second factor in cell selection, minimization of total axonal length, lowering metabolic demand in the surviving cells, will assist their survival [40], and favour the evolution of a small-world configuration [7,50].

### 4.1. Columnar versus Noncolumnar Cortex

It is useful to first explain how, in this model, the difference between columnar versus noncolumnar cortex comes about. Simulation of cortical development [51] shows small world selection and selection for maximum synchrony can be in conflict. It is the relative length of long and short axon neurons included in the simulation that determines whether clearly columnar, or apparently diffuse, non-columnar organization results. Suppose two populations of cortical neurons, with axonal tree distributions

(11) $$\rho_{\alpha }=N_{\alpha }\lambda_{\alpha }\exp\left[-\lambda_{\alpha }x\right]$$

(12) $$\rho_{\beta }=N_{\beta }\lambda_{\beta }\exp\left[-\lambda_{\beta }x\right]$$

where $\rho_{\alpha }\;(x),\rho_{\beta }(x)$ are respective normalized densities of the axonal trees of long-axon, α cells, and short-axon, β cells, as a function of distance, x, from their cell somas. The fraction of presynapses generated by the two cell types are $N_{\alpha },N_{\beta }$, and $\lambda_{\alpha },\lambda_{\beta }$, are their axonal inverse length constants.

Bidirectional connection density, $\rho_{\alpha +\beta }$, for all cells would be a maximum if

(13) $$\rho_{\alpha +\beta }\left(x\right)=N_{\alpha }\lambda_{\alpha }\exp\left[-\lambda_{\alpha }x\right]+N_{\beta }\lambda_{\beta }\exp\left[-\lambda_{\beta }x\right]$$

whereas density of connection in an ultra-small world network [52] where inter-soma distance is surrogate for increasing order of neighbour separation, is given by ${\rho_{\alpha +\beta }\left(x+k\right)}^{-2}$. Thus, disparity of connection density, Δ(x), of an ultra-small world system and that of the axonal trees of α and β cells is at best

(14) $$\Delta \left(x\right)={\left(x+k\right)}^{-2}-\left(N_{\alpha }\lambda_{\alpha }\exp\left[-\lambda_{\alpha }x\right]+N_{\beta }\lambda_{\beta }\exp\left[-\lambda_{\beta }x\right]\right)$$

and competitive processes maximizing synchrony (see below) force further departures in separation of cell bodies from the ultra-small optimum.

**Figure 3:**
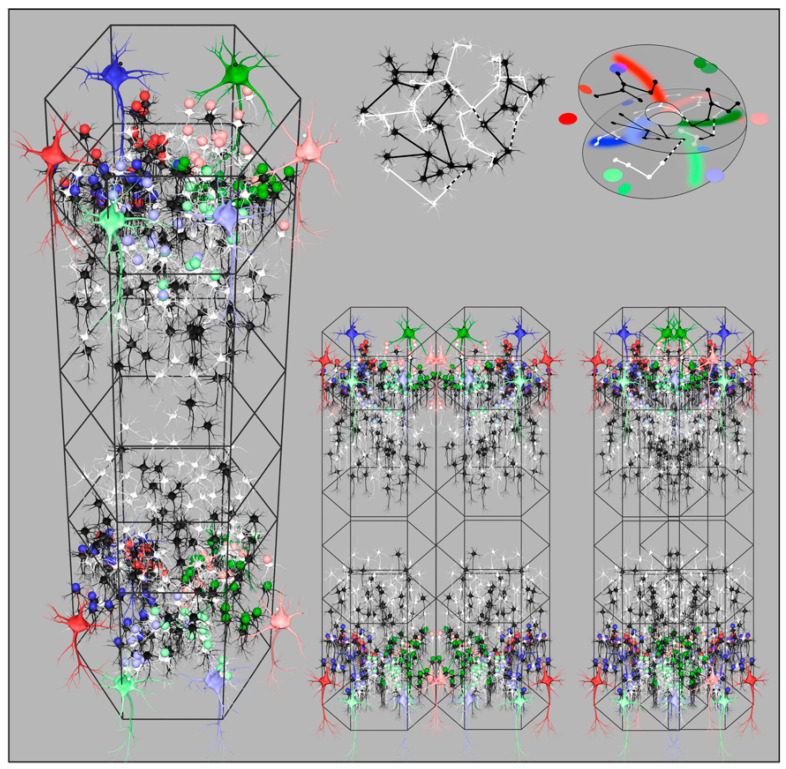
Organization of cortical columns. Left: Reconstruction showing disposition of cells and synapses for maximum synchrony in a surface-oblique view of a column. Large coloured neurons represent superficial patch cells. Black and white smaller cells are local short-axon excitatory cells. Small coloured spheres represent presynapses efferent from patch cells of the same colour. (Return bidirectional synaptic connections not shown.). Similar synaptic self-organization is shown in the deeper layers of the column. Right top left: A subset of local cells from the reconstruction are shown in isolation, indicating the way that interpenetration of networks of local cells is a consequence of sparsity of connection. Black and white colouration is arbitrary other than to indicate the interweaving. Occasional cross-links, shown as dashed black and white, bridge the sparse networks, and result in amplification of synchrony in closed loops. Right top right: An abstract representation of the networks right top, showing the cells as arrayed in a closed loop configuration analogous to a Mobius strip. Coloured dots and swaths of colour show how presynapses from patch cells are deployed to maximize co-resonance between local and patch cells. Right bottom: Two views of adjacent columns. On the left is the arrangement in columnar neocortex. Columns abut, but do not overlap, and synaptic organization is mirrored between columns. On the right, the arrangement in noncolumnar cortex. The two columns are interpenetrating, permitted by the sparsity of connections, and there is no difference in synaptic organization—but small-world organization has predominated over maximum synchrony organization.

Simulations of cortical growth presumed that the axonal tree lengths are genetically determined and that the numbers of cells in the two populations are selected so as to optimize both synchrony and small world connectivity. For higher and more equal values of $\lambda_{\alpha }$ and $\lambda_{\beta }$ ultra-small world order is most closely approximated, therefore predominates, and columnar definition is not apparent. Where $\lambda_{\alpha }\ll \lambda_{\beta }$, maximization of synchrony among the numerous short-axon neurons is the predominant influence, and clearly columnar organization results. The loss of definition in the noncolumnar instances arises from the merging and inter-weaving of cell networks, made possible by the sparsity of synaptic connectivity. Whether apparently columnar or diffuse, simulations show that the same patterns of synaptic connections best maximizing synchrony are present, but are organized in interdigitated overlapping systems where small-world organization has predominated. As is later explained, Figure 3, bottom right, illustrates the way this merging takes place.

It is emphasized that in the following account, the description of emerging patterns of synaptic connection is to considered general throughout cortex, although comparison with the clearly columnar visual cortex (V1), for many years the focus of experimental study, enables more direct comparison between theory and experiment. 

### 4.2. Early Embryonic Development

At the earliest stage developing synaptic connections are initially random, and polysynaptic pathways between any two neurons develop as cells and synapses proliferate, bringing about polysynaptic flows that are roughly bidirectionally symmetrical between all cells, so synchrony is early apparent in developing cells, as they begin to associate into small world systems [46]. Bidirectional monosynaptic connections begin to develop, preferentially selected out of the polysynaptic flow between neurons, further increasing magnitude of synchrony. 

As previously described, for simplicity we treat distribution of axonal lengths in the developing cells as two populations—one of excitatory cells with long axons, and a short axon population of mixed excitatory and inhibitory cells [53].

At a distance, X, from their cell bodies, the population density of the axonal trees of the short-axon and long axon cell populations are equal.

(15) $$X=\frac{-ln\left(\frac{N_{\alpha }\lambda_{\alpha }}{N_{\beta }\lambda_{\beta }}\right)}{\lambda_{\beta }{-\lambda }_{\alpha }}$$

The short-axon, β, cells whose axonal density is greatest at short range, preferentially form densest connections with each other at distances less than X, clustering into columnar-like systems. The long axon, α, cells form preferential connections in patches where their cell bodies are closely situated, and because of competitive exclusion by β cell synapse formation, form other preferential long-range connections at distances greater than X—so that patches of α cells form with skipping connections at lengths that are multiples of X, in a grid with edges of length X, enclosing clusters of short-axon cells. This reproduces the superficial patch cell network. 

The long axon cells and short axon cells exchange bidirectional monosynaptic connections at distance X. The upshot is that within each cluster the short-axon cells and their surrounding patches of long-axon cells project synapses to each other 1:1, maximizing synchrony by creating swaths of connection in arcs of a circle (in two dimensions) or segments of a spherical surface (in three dimensions) of radius X. Again because of synaptic sparsity, the formation of 1:1 maps is not confined to a simple Euclidean projection, but can project from the clusters of α cells to separate, interpenetrating, parts of the enclosed β networks as the Rheimann projection that will best maximize joint synchrony. Positions in the α-cell network can be considered as global positions in the cortical area, and designated complex number positions, P, while positions in any of the local β-cell clusters are designated p. (The complex plane positions may be further generalised to positions in three dimensions, as required.). As bidirectional monosynaptic connections emerge, they result in global-to-local maps of the form

(16) $$P↔p\;where\;p=\pm \sqrt{-1}k\frac{{\left(P-p_{0}\right)}^{n}}{{\left|P-p_{0}\right|}^{n-1}}+p_{0}$$

${\left(P-p_{0}\right)}^{n}/{\left|P-p_{0}\right|}^{n-1}$ describes angular multiplication by n in the projection from P to p. The factor $\sqrt{-1}k$ defines the rotation by 90 degrees and scale of the projection created by the arcs of synapses. Chirality is shown + or −, and $p_{0}$ is the centre of a short-axon β cell cluster. This is a mirror-mapping in a topological sense—the global field being reflected in each local map. Figure 3 left shows a reconstruction of these synaptic projections in the upper, and in the lower, layers of a developing column. 

The value of n also represents the number of turns about the β cell cluster centre made by sparse and interpenetrating β cell networks before they form a closed self-exciting system, and the global-to-local projection must match the closed loop conformation in the form best maximizing synchrony. The projection of α cells to β cells from diametrically opposite sides of a local map, each at range X, forces their synapses to be deployed in arcs radiating from the local maps center—either deployed on opposite sides of the map center—in which case n=1—or both radiating from the center on the same side—in which case n=2. The n=1 case is a simple Euclidean mapping, whereas n=2 is a mapping analogous to the mapping of a plane onto a Mobius strip. The latter arrangement permits greater total synchrony by dint of the longer chains of connection among the sparse, but cross-connected, β cell networks. Angles in the global field from 0−π are mapped locally from 0−2π in the plane view of the column, while global angles from π−2π are also mapped (on a separate mesh of cells) from 0−2π in the same view, creating the form of an orientation preference singularity. Figure 3 top right shows how connections in the interpenetrating nets of sparsely connected cells can be construed in this way.

By forming mirror symmetry arrangement of adjacent local maps, homologous positions in the projections from the global map are brought into highest contiguity—thus enabling them to form connections further maximizing their joint synchrony. That is

(17) $$p_{A}↔p_{B}\;where\;p_{A}=+\sqrt{-1}k\frac{{\left(P-p_{0A}\right)}^{2}}{\left|P-p_{0A}\right|}+p_{0A}\;\;and\;p_{B}=-\sqrt{-1}k\frac{{\left(P-p_{0B}\right)}^{2}}{\left|P-p_{0B}\right|}+p_{0B}$$

A and B indicate adjacent local maps (columns). The arrangement may be discrete and columnar, or the adjacent maps may themselves be interpenetrating to variable degree in noncolumnar cortex, as shown bottom right in Figure 3—synchrony will still be maximized.

Similarly, maps can form at different depths on the six-layered cortex. As these form in layers, each similarly oriented with regard to the surrounding global map, they are arranged in mirror symmetry in the axis of cortical depth.

Experimental findings explained by this model include patch cell clustering and interpatch order, the organization of orientation preference (OP) in monocular areas of V1 including OP singularities, linear zones, and saddle points, and in binocular ocular dominance (OD) columns—also explaining the “like-to-like” connections made by patch cells to short-axon cells with common OP preference in separate local maps. 

A critical test of this explanation of the organization of OP maps [53] was passed in the simulation of variation of OP when measured using moving visual lines with differing angle of attack, line length, and stimulus speed [54]—a finding explained by lag times of conduction in lateral intracortical connections. This distinguishes the present model from feedforward, self-organizing map, and dimension reduction models of OP organization. Although the two contrasted model types are compatible, pure feedforward supposes only fixed feature representations and does not include effects of lateral contextual interactions. 

A separate consideration applies to formation of mirror assemblies maximizing joint synchrony as cortico-cortical connections develop, creating inter-area linkage. Cortico-cortical projections form U-shaped loops in cortical white matter, projecting from one cortical area to its neighbours with mirror symmetry, and with subsequent onward projections to further cortical areas creating observable recurrent reversals of map chirality [55]. This can be accounted for as a simple consequence of the form of the fibre projections [56] although the complexity of interareal connections and hierarchies obscures the effect in some cases.

**Figure 4:**
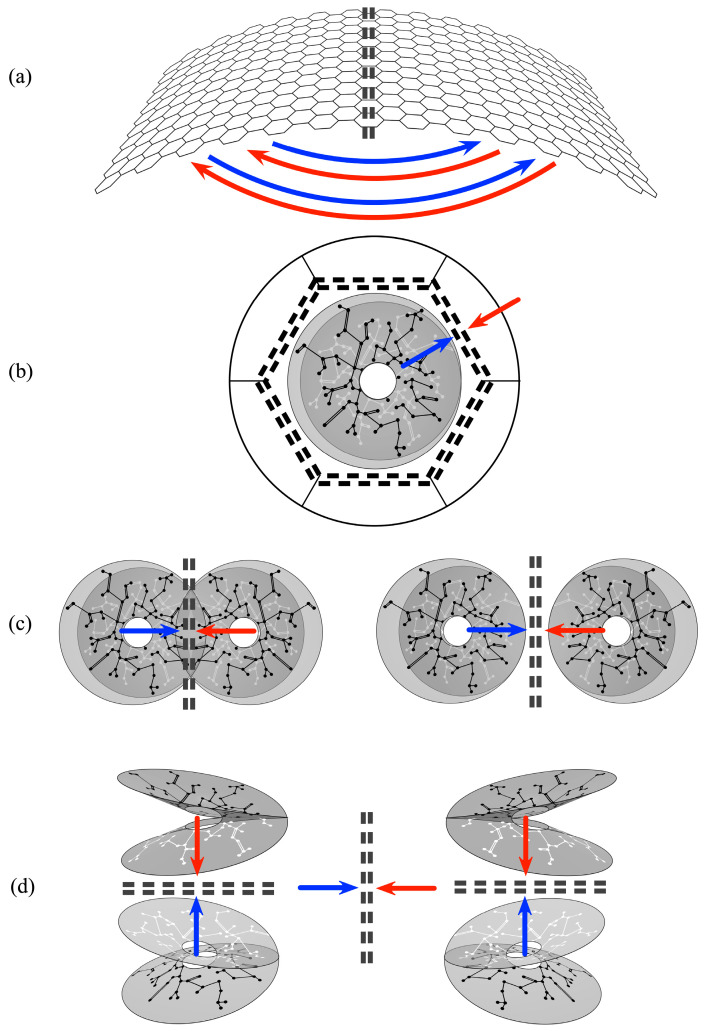
Summary representation of all the ways mirror representations arise. Double black dashed lines indicate lines of mirror symmetry, and putative Markov blankets. Red and blue arrows indicate presynaptic flows toward lines of symmetry. (**a**) Cortico-cortical and Inter-areal connections. Their U-shaped form projects each cortical area to its neighbours with mirror symmetry. (**b**) Each local map interacts with the global map with (topological) mirror symmetry, as the local short-axon neurons exchange flux with the surrounding cortex via the patch cell system. (**c**) Local cell groups interact with adjacent groups of opposite chirality—whether the groups interpenetrate, abut, or are further separated. (**d**) Within every column mirror symmetry is generated between layers, while also able to interact laterally with other mirrored systems.

Thus a multitude of mirror systems can tile the cortex, as adjacent columns, as interpenetrating sparse systems equivalent to columns, or as systems separated but interconnected by cortico-cortical connections. They can be mirrored in layers of cortical depth, with each layer laterally mirrored. They form mirrors between scales, as the patch system projects to each column or its non-columnar equivalent, and as mirrors between entire cortical areas. (Figure 4). These alternative ways in which mirrors can be arranged form the set of topographies that can be created within neocortex, each corresponding to the topology of the theoretical unit in Figure 2.

### 4.3. Later Embryonic and Early Antenatal Development

Early in antenatal life sensory afferents reach the cortex [57] and eventually impose complicated temporal structure on the inputs to the cortex, replacing the earlier stochastic exchanges. The radially symmetric mirror structures are now able to act as a scaffold upon which spatiotemporal images can be stored and read out.

#### 4.3.1. Spatiotemporal Images

As an external stimulus is imposed upon the cortex, signals relayed intracortically from the global to the local scales, arrive in neighbouring macrocolumns within a short epoch. Concurrent arrivals at closely situated neurons generated from different positions and different times in the global field are able to promote synchrony and secondary formation of synapses between the local cells. It can be shown [58] that this can result in the storage in the local map of the representation of a moving image $P,(t-\frac{\left|P-p\right|}{\upsilon })\to \;p(t)$. This provides the basis of variation of OP with stimulus velocity and orientation mentioned above. Representations formed in this manner can differ in the information about the object represented. Information from widespread positions in the global field would better represent movement than shape, and from positions closely situated in the global field, the shape of the object. This may account for representations higher in the cortical hierarchy specializing in differing types of visual information—the dorsal and ventral visual streams [59]. 

Chains of such images would store more complicated sequences, and in motor cortex, reversal of the processes could be read out as spatiotemporal motor outputs.

#### 4.3.2. Coupled Spatial Eigenmodes, Spatial and Temporal Frequency Preferences

The process generating spatiotemporal images is equivalent to the generation of coupled spatial eigenmodes, and explains other response preferences of V1 neurons [59]. Signals from positions in the global field circumferentially arrayed with respect to local maps generate a high frequency response in the local cells, in contrast to the lower frequency of responses elicited from radially positioned inputs. Synchronous fields thus generated are preferentially tuned to high frequencies and arrayed circumferentially within local maps, or tuned to low frequencies and arrayed radially. Adjacent circumferential high frequency domains are readily coupled by unidirectional excitatory couplings, as are adjacent radial low frequency domains—but the orthogonal disposition and poor frequency matching of high and low frequency domains leads them to be mutually antagonistic via their inhibitory surrounds. These properties account for the spatial (SFP) and temporal (TFP) frequency preferences of local cells [17,60]. High SFP cells (HSFP) occur most commonly in linear zones near the circumferential perimeter of macrocolumns. Low SFP (LSFP) zones are more scattered and radially located. At OP singularities either an HSFP domain or an LSFP domain is located-interpreted as competitive conflict forcing one or other outcome. Temporal frequency preferences (TFP) are accounted for along with SFP, since it is known that TFP = stimulus velocity × SFP [61]—as expected for intracortical laterally spreading signals. HSFP/HTFP and LSFP/LTFP zones thus appear to reveal the existence of coupled spatial eigenmodes on each macrocolumn.

Adjacent macrocolumns must receive inputs from the global field that are from the same stimulus, translated in space and time. Since the scaffold structure of each macrocolumn approximates a mirror reflection of its neighbours, adjacent macrocolumns could interact with each other as envisaged in Figure 2, with the line of junction acting as a Markov blanket. By reaching a co-synchronous stable exchange, they would have abstracted and stored wider general characteristics of the stimulus object’s shape and movement.

## 5. Conclusions

### 5.1. The Logical Structure of Our Argument

We have shown that application of the free energy principle to a simple but realistic neural field leads to a theoretical unit of self-organization, constructed of mirrored assemblies of synaptic connections, and separated by a Markov blanket. On the other hand, simulations of development in the neocortex lead to a compatible outcome—with provisos. The conflicting demands of maximized synchrony versus small-world organization mean that the outcome of growth simulations is expressed in the simplest topographic relation to the theoretical unit only in columnar cortex. The sparsity of all neuronal connections accounts for the way a single unit of synaptic organization can be masked by the interpenetration of separated networks. In a related way, sparsity of connection accounts for the form of OP singularities and the Mobius strip-like form that the mirrored assemblies must take. 

This means that our argument is limited by the fact that the ubiquity of paired mirror representations is inferred rather than directly demonstrated, and even in the relatively clear case of mirror organization of OP seen in OD columns, this is an interpretation of the underlying connections rather than direct visualization. However, the growth model has wide explanatory power, greater than any preceding model, for findings in the visual cortex—notably accounting for the topographic organization of OP, SFP, TFP, and like-to-like connections, and also reproducing the dynamic variations of OP with object speed and angle. It explains why OP maps are apparent at birth, since emergence of these structures requires only noise-like driving, and only a radially symmetrical structure appears at that stage. Likewise, it also accounts for the results of postnatal visual deprivation, since it requires ongoing post-natal learning to overwrite the radially organized antenatal scaffold. Therefore there can be some confidence in the growth model’s validity, and the growth simulation outcomes show that it is logically consistent to extend the model to the neocortex in general.

### 5.2. The Properties of the Theoretical Unit—Internal Markov Blankets

The theoretical unit, derived directly from the free energy principle, makes explicit an extra property not obvious from the growth model alone—the development of a Markov blanket between each pair of mirror-ordered connections. Opposed signals are not brought directly into matching interaction, but their cumulative effects on eigenmode coupling within each of the mirror assembly pair are brought to excitatory/inhibitory balance at the line of mirror junction, and thus mutual information between the mirror pairs is maximized. This has major functional implications, introducing local stabilization and the interplay of extensive co-synchrony with prediction error minimization throughout the neural field, at all scales and in cortical depth. Error minimization is not restricted to particular special systems, as in the canonical model of error minimization. Overall, error minimization proceeds in a fully distributed fashion and provides a universal mechanism for the abstraction and storage of common features in cortical interactions at all scales. 

This can be illustrated by explanation of the enigmatic relationship of OD columns to binocular fusion in different species. In the more straightforward case in which OD columns are present, laterally adjacent OD columns, interacting via a Markov blanket while each receiving an input from the visual field from opposite eyes, can achieve maximum mutual information with each other, while similarly interacting with mirror assembles higher and lower within each column. Maximization of their joint mutual information utilizes effects of perspective to create a representation equivalent to a 3D image. Yet the synaptic organization achieving this effect does not depend on specific columnar order, and could exist perfectly well if the cell bodies composing the columns were intermingled. It is the synaptic topology that is important. Thus species without cortical columns in V1 can still have 3D vision, because they have separated inputs from each eye in OD- like conformation.

### 5.3. Generalization to Development and Function beyond the Neocortex

The developmental growth model is cast in terms of neocortical self-organization, with long-range excitatory connections and simplified intrinsic axonal ranges emphasized. This begs the question of integration of neocortical and subcortical systems not only as pathways of sensory and motor interactions with environment, but in the regulation of cortical arousal and attentional focus, and of reinforcement. These aspects have been given brief attention in regard to the growth model [62]. 

A further question is whether the development of mirror symmetric synaptic systems with intervening Markov blankets may be applicable more widely, to neurogenesis in general—and more particularly might be applicable to the paleo- and archicortical progenitors of the neocortex in the structural model. A wider unification by amalgamation with analyses of limbic neocortical relations [5,6] might then be possible. As the cortex emerges by differentiation from cells of limbic origin, during its growth it might be brought, by the same process of predictive error minimization, into harmony with the developing limbic and subcortical systems, while concurrently more direct exchanges of neocortex and environment via major sensory and motor pathways develop.

There appears no restriction to developing further growth models along these lines. Oscillation occurs, and can be modelled in other brain systems with wholly different synaptic architecture and fiber ranges—e.g., olfactory cortex [63]. The theoretical account of development of mirror systems with intervening mirror blankets is wholly general, subject only to the listed constraints, so specifics of connectivity will affect the topographies of connection, not their topology. It may be assumed that the same selective processes could operate among neural precursors with widely different genetic variations in available cell types. Although the growth model depends upon selection by apoptosis of synapses and cell positions maximizing zero-lag synchrony, it is unclear whether this is the only selection that might apply widely in the brain—or, indeed, in the neocortex itself. The maximization of zero-lag synchrony is not a unique pathway to minimum variational free energy. In other circumstances paired mirror systems organized into limit cycles, or chaotic attractor systems, are theoretically possible, and could occur in any neural system in which prediction error minimization was an essential attribute. 

### 5.4. Testing and Cellular Mechanisms 

The growth model is subject to further testing on a rather grand, but definitive level. Connectivity analysis in both columnar and noncolumnar cortex ought to establish that linkages of patch cells and short axon cell clusters are similar in both types-discrete in columnar cortex, and overlapping in noncolumnar cortex. At the same detailed microscopic level, it should be possible to demonstrate that within short-axon clusters like-to-like connections terminate in a Mobius-like manner, on the interpenetrating and intertwined short-axon local cell networks.

Integration of this model with mechanisms of anti-Hebbian plasticity, synaptogenesis, apoptosis, and the role of neural energetics, needs to be further demonstrated or disproved, when further advances in these fields permit. Finally, it may be remarked that large-scale chip emulations of neurons in mirror arrays may be practicable, and might then provide an anatomically realistic framework in which to explore unsupervised learning.

## Data Availability

The original contributions presented in the study are included in the article, further inquiries can be directed to the corresponding author.

## References

[1] Barbas H. (1986). Pattern in the laminar origin of corticocortical connections. J. Comp. Neurol..

[2] Barbas H., Rempel-Clower N. (1997). Cortical structure predicts the pattern of corticocortical connections. Cereb. Cortex.

[3] Barbas H. (2015). General cortical and special prefrontal connections: Principles from structure to function. Annu. Rev. Neurosci..

[4] García-Cabezas M., Zikopoulos B., Barbas H. (2019). The structural model: A theory linking connections, plasticity, pathology, development and evolution of the cerebral cortex. Anat. Embryol..

[5] Tucker D.M., Luu P. (2023). Adaptive control of functional connectivity: Dorsal and ventral limbic divisions regulate the dorsal and ventral neocortical networks. Cereb. Cortex.

[6] Luu P., Tucker D.M. (2023). Continuity and change in neural plasticity through embryonic morphogenesis, fetal activity-dependent synaptogenesis, and infant memory consolidation. Dev. Psychobiol..

[7] Markov N.T., Misery P., Falchier A., Lamy C., Vezoli J., Quilodran R., Gariel M.A., Giroud P., Ercsey-Ravasz M., Pilaz L.J. (2011). Weight consistency specifies regularities of macaque cortical networks. Cereb. Cortex.

[8] Vezoli J., Magrou L., Goebel R., Wang X.-J., Knoblauch K., Vinck M., Kennedy H. (2020). Cortical hierarchy, dual counterstream architecture and the importance of top-down generative networks. NeuroImage.

[9] Aparicio-Rodríguez G., García-Cabezas M. (2023). Comparison of the predictive power of two models of cortico-cortical connections in primates: The distance rule model and the structural model. Cereb. Cortex.

[10] Campbell K. (2005). Cortical neuron specification: It has its time and place. Neuron.

[11] Muir D.R., Da Costa N.M.A., Girardin C.C., Naaman S., Omer D.B., Ruesch E., Grinvald A., Douglas R.J. (2011). Embedding of cortical representations by the superficial patch system. Cereb. Cortex.

[12] Muir D.R., Douglas R.J. (2010). From neural arbors to daisies. Cereb. Cortex.

[13] Martin K.A.C., Roth S., Rusch E.S. (2014). Superficial layer pyramidal cells communicate heterogeneously between multiple functional domains of cat primary visual cortex. Nat. Commun..

[14] Molnar Z., Rubenstein J.L.R., Rakic P. (2013). Cortical columns. Comprehensive Developmental Neuroscience: Neural Circuit Development and Function in the Brain.

[15] Bosking W.H., Zhang Y., Schofield B., Fitzpatrick D. (1997). Orientation selectivity and the arrangement of horizontal connections in tree shrew striate cortex. J. Neurosci..

[16] Rockland K.S., Lund J.S. (1983). Intrinsic laminar lattice connections in primate visual cortex. J. Comp. Neurol..

[17] Issa N.P., Trepel C., Stryker M.P. (2000). Spatial frequency maps in cat visual cortex. J. Neurosci..

[18] Girman S.V., Sauvé Y., Lund R.D. (1999). Receptive field properties of single neurons in rat primary visual cortex. J. Neurophysiol..

[19] Wiesel T.N., Hubel D.H. (1974). Ordered arrangement of orientation columns in monkeys lacking visual experience. J. Comp. Neurol..

[20] Blakemore C., Van Sluyters R.C. (1974). Reversal of the physiological effects of monocular deprivation in kittens: Further evidence for a sensitive period. J. Physiol..

[21] Obermayer K., Blasdel G. (1993). Geometry of orientation and ocular dominance columns in monkey striate cortex. J. Neurosci..

[22] Takahata T., Miyashita M., Tanaka S., Kaas J.H. (2014). Identification of ocular dominance domains in new world owl monkeys by immediate-early gene expression. Proc. Natl. Acad. Sci. USA.

[23] Horton J.C., Adams D.L. (2005). The cortical column: A structure without a function. Philos. Trans. R. Soc. B Biol. Sci..

[24] Friston K. (2002). Functional integration and inference in the brain. Prog. Neurobiol..

[25] Friston K., Friston K. (2005). A theory of cortical responses. Philos. Trans. R. Soc. B Biol. Sci..

[26] Friston K., Friston K. (2010). The free-energy principle: A unified brain theory?. Nat. Rev. Neurosci..

[27] Friston K. (2010). Is the free-energy principle neurocentric?. Nat. Rev. Neurosci..

[28] Friston K. (2022). Maps and territories, smoke, and mirrors. Behav. Brain Sci..

[29] Friston K., Ao P. (2012). Free Energy, Value, and Attractors. Comput. Math. Methods Med..

[30] Friston K., Levin M., Sengupta B., Pezzulo G. (2015). Knowing one’s place: A free-energy approach to pattern regulation. J. R. Soc. Interface.

[31] Friston K., Thornton C., Clark A. (2012). Free-energy minimization and the dark-room problem. Front. Psychol..

[32] Friston K.J., Parr T., Yufik Y., Sajid N., Price C.J., Holmes E. (2020). Generative models, linguistic communication and active inference. Neurosci. Biobehav. Rev..

[33] Buckley C.L., Kim C.S., McGregor S., Seth A.K. (2017). The free energy principle for action and perception: A mathematical review. J. Math. Psychol..

[34] Constant A. (2021). The free energy principle: It’s not about what it takes, it’s about what took you there. Biol. Philos..

[35] Ramstead M.J.D., Sakthivadivel D.A.R., Heins C., Koudahl M., Millidge B., Da Costa L., Klein B., Friston K.J. (2022). On Bayesian Mechanics: A Physics of and by Beliefs. Interface Focus.

[36] Beim Graben P., Pinotsis D., Saddy D., Potthas R. (2008). Language processing by dynamic fields. Cogn. Neurodyn..

[37] Kirchhoff M., Parr T., Palacios E., Friston K., Kiverstein J., Michael K., Thomas P., Ensor P., Karl F., Julian K. (2018). The Markov blankets of life: Autonomy, active inference and the free energy principle. J. R. Soc. Interface.

[38] Luu P., Tucker D.M., Friston K. (2023). From active affordance to active inference: Vertical integration of cognition in the cerebral cortex through dual subcortical control systems. Cereb. Cortex.

[39] Bastos A.M., Usrey W.M., Adams R.A., Mangun G.R., Fries P., Friston K.J. (2012). Canonical microcircuits for predictive coding. Neuron.

[40] Vergara R.C., Jaramillo-Riveri S., Luarte A., Moënne-Loccoz C., Fuentes R., Couve A., Maldonado P.E. (2019). The energy homeostasis principle: Neuronal energy regulation drives local network dynamics generating behavior. Front. Comput. Neurosci..

[41] Perin R., Berger T.K., Markram H. (2011). A synaptic organizing principle for cortical neuronal groups. Proc. Natl. Acad. Sci. USA.

[42] Song S., Sjöström P.J., Reigl M., Nelson S., Chklovskii D.B. (2005). Correction: Highly nonrandom features of synaptic connectivity in local cortical circuits. PLoS Biol..

[43] Izhikevich E.M., Desai N.S. (2003). Relating STDP to BCM. Neural Comput..

[44] Keck T., Toyoizumi T., Chen L., Doiron B., Feldman D.E., Fox K., Gerstner W., Haydon P.G., Hübener M., Lee H.-K. (2017). Integrating Hebbian and homeostatic plasticity: The current state of the field and future research directions. Philos. Trans. R. Soc. B Biol. Sci..

[45] Chapman C.L., Wright J.J., Bourke P.D. (2002). Spatial eigenmodes and synchronous oscillation: Co-incidence detection in simulated cerebral cortex. J. Math. Biol..

[46] Downes J.H., Hammond M.W., Xydas D., Spencer M.C., Becerra V.M., Warwick K., Whalley B.J., Nasuto S.J. (2012). Emergence of a small-world functional network in cultured neurons. PLoS Comput. Biol..

[47] Hollville E., Romero S.E., Deshmukh M. (2019). Apoptotic cell death regulation in neurons. FEBS J..

[48] Heck N., Golbs A., Riedemann T., Sun J.-J., Lessmann V., Luhmann H.J. (2007). Activity-dependent regulation of neuronal apoptosis in neonatal mouse cerebral cortex. Cereb. Cortex.

[49] Sang I.E.W.F., Schroer J., Halbhuber L., Warm D., Yang J.-W., Luhmann H.J., Kilb W., Sinning A. (2021). Optogenetically controlled activity pattern determines survival rate of developing neocortical neurons. Int. J. Mol. Sci..

[50] Bassett D.S., Bullmore E. (2006). Small-world brain networks. Neurosci..

[51] Wright J.J., Bourke P.D. (2016). Further Work on the shaping of cortical development and function by synchrony and metabolic competition. Front. Comput. Neurosci..

[52] Cohen R., Havlin S. (2003). Scale-free networks are ultrasmall. Phys. Rev. Lett..

[53] Wright J.J., Bourke P.D. (2003). On the dynamics of cortical development: Synchrony and synaptic self-organization. Front. Comput. Neurosci..

[54] Basole A., White L.E., Fitzpatrick D. (2003). Mapping multiple features in the population response of visual cortex. Nature.

[55] Sereno M.I., Dale A.M., Reppas J.B., Kwong K.K., Belliveau J.W., Brady T.J., Rosen B.R., Tootell R.B.H. (1995). Borders of multiple visual areas in humans revealed by functional magnetic resonance imaging. Science.

[56] Konkle T. Emergent organization of multiple visuotopic maps without a feature hierarchy. bioRxiv.

[57] Espinosa J.S., Stryker M.P. (2012). Development and plasticity of the primary visual cortex. Neuron.

[58] Wright J.J., Bourke P.D. (2022). Unification of free energy minimization, spatiotemporal energy, and dimension reduction models of V1 organization: Postnatal learning on an antenatal scaffold. Front. Comput. Neurosci..

[59] Sheth B.R., Young R. (2016). Two visual pathways in primates based on sampling of space: Exploitation and exploration of visual information. Front. Integr. Neurosci..

[60] Issa N.P., Rosenberg A., Husson T.R. (2008). Models and measurements of functional maps in V1. J. Neurophysiol..

[61] Baker C.L. (1990). Spatial- and temporal-frequency selectivity as a basis for velocity preference in cat striate cortex neurons. Vis. Neurosci..

[62] Wright J.J., Bourke P.D. (2023). The mesoanatomy of the cortex, minimization of free energy, and generative cognition. Front. Comput. Neurosci..

[63] Liljenström H. (1991). Modelling the dynamics of olfactory cortex using simplified network units and realistic architecture. Int. J. Neural Syst..

